# Immunotactoid glomerulopathy – an enigmatic case in the setting of nodal marginal zone lymphoma and systemic sclerosis sine scleroderma

**DOI:** 10.1186/s12882-022-02730-w

**Published:** 2022-03-15

**Authors:** Mohamed Wael Mohamed, Mariam Al-Hammadi, Ali Mohammad Hussein, Daher Alarab, Hisham Ahmad Albreak, Mohammad Fahim Tungekar, Balaji Dandi

**Affiliations:** 1 Internal Medicine Department, King Hamad University Hospital, Muharraq, Kingdom of Bahrain; 2 Nephrology Unit, King Hamad University Hospital, Muharraq, Kingdom of Bahrain; 3 Uranologics Ltd, London, United Kingdom & Expressmed Laboratories, Manama, Kingdom of Bahrain

**Keywords:** Immunotactoid glomerulopathy, Nephrotic syndrome, Nodal marginal zone lymphoma, Renal biopsy, Rituximab, Systemic sclerosis sine scleroderma, Case report

## Abstract

**Background:**

Immunotactoid Glomerulopathy (ITG) is an exceedingly rare type of glomerulopathy characterised by distinctive electron microscopic features. ITG has been linked to lymphoproliferative or autoimmune disorders. The clinical manifestations are diverse including nephrotic syndrome (NS), haematuria, acute kidney injury and end stage renal failure (ESRD). We present a case with a stage 3 Nodal Marginal Zone Lymphoma (NMZL) and systemic sclerosis sine scleroderma (SSSS), where the evolution of ITG was documented in 2 renal biopsies 19 months apart. To the best of our knowledge, no cases have been reported linking ITG to NMZL. Furthermore, there is only one non-peer reviewed report linking ITG to scleroderma. We discuss the implications of our findings and highlight the satisfactory management of the case.

**Case presentation:**

A 79-year-old female with history of systemic sclerosis sine scleroderma and stage 3 NMZL presented with acute kidney injury and NS on a background of chronic kidney disease. Her first kidney biopsy showed a diffuse proliferative glomerulonephritis and her serum protein electrophoresis showed no abnormalities. She was managed satisfactorily with conservative measures. She returned 19 months later with features of fluid overload, increasing proteinuria and rising serum creatinine. A repeat serum protein electrophoresis showed excess free kappa light chains and ITG was detected in the repeat kidney biopsy. Her kidney function and proteinuria showed a good and sustained response to rituximab administered after the second biopsy.

**Conclusion:**

ITG is a rare type of glomerulopathy, associated with underlying haematological malignancies and autoimmune disorders that may result in ESRD.

Rituximab is one of the effective agents used in the management of ITG with haematological malignancies.

## Background

Glomerular diseases can rarely be associated with organised deposits that are broadly divided into Congo Red positive (amyloid) and Congo Red negative (non-amyloid) [1–4]. The Congo Red negative conditions are extremely rare and are mainly divided into fibrillary and Immunotactoid Glomerulopathy (ITG) based on fibril morphology including thickness of the fibrils [3, 4].

ITG can cause several manifestations including proteinuria, haematuria, hypertension (HTN), acute kidney injury and may lead to end stage renal disease (ESRD) [2, 3]. ITG has been linked most commonly with haematological malignancies particularly low grade B-cell lymphomas and myeloma [2–5]. Thus, a North American study published in 2020 that looked at 73 cases of ITG found that the most common cause was an underlying haematological disorder most commonly a B-cell lymphoma or myeloma, however 10 patients (14%) had an underlying autoimmune condition [2]. A French study of 27 cases of ITG with monoclonal gammopathy, had underlying low grade B-cell lymphomas and myelomas in 18 cases (67%) [3]. Nodal Marginal Zone Lymphoma (NMZL) represent 1% of all non-Hodgkin’s lymphomas and 10% of all Marginal Zone Lymphomas (MZL) and may be associated with monoclonal gammopathy [6]. The association of MZL to autoimmune diseases is well-recognised, as is its rare occurrence in systemic sclerosis [7, 8].

We report a patient who presented with acute on chronic kidney disease and nephrotic syndrome (NS) who was eventually diagnosed as ITG with concurrent NMZL and systemic sclerosis sine scleroderma (SSSS).

## Case presentation

A 79-year-old female initially presented with acute kidney injury with proteinuria of 5 g/24 hours, reduced urine output and serum creatinine of 97 μmol/L from a baseline of 57 μmol/L. She was a known case of SSSS with gastroesophageal reflux disease and pulmonary involvement with positive ANA 1:100 (dotted nucleoplasm pattern), anti-centromere protein B antibody level of 490μg/mL [reference range (RR) 0-10 μg/mL] and a positive nail fold capillaroscopy test. Biopsy of her enlarged left submandibular lymph node in April 2018 showed a CD20, CD79a and BCL2 positive nodal marginal zone lymphoma. Subsequent CT and PET Scans assessed it as a stage 3 NMZL on the basis of cervical, mediastinal, abdominal and inguinal nodes involvement.

Serum protein electrophoresis/immunofixation was negative for monoclonal gammopathy and C4 levels were low. Other co-morbidities included atrial fibrillation and uncontrolled HTN for which she was prescribed four antihypertensive medications. Serology for cryoglobulins, hepatitis B virus, hepatitis C virus, and human immune deficiency virus was negative.

**Fig. 1:**
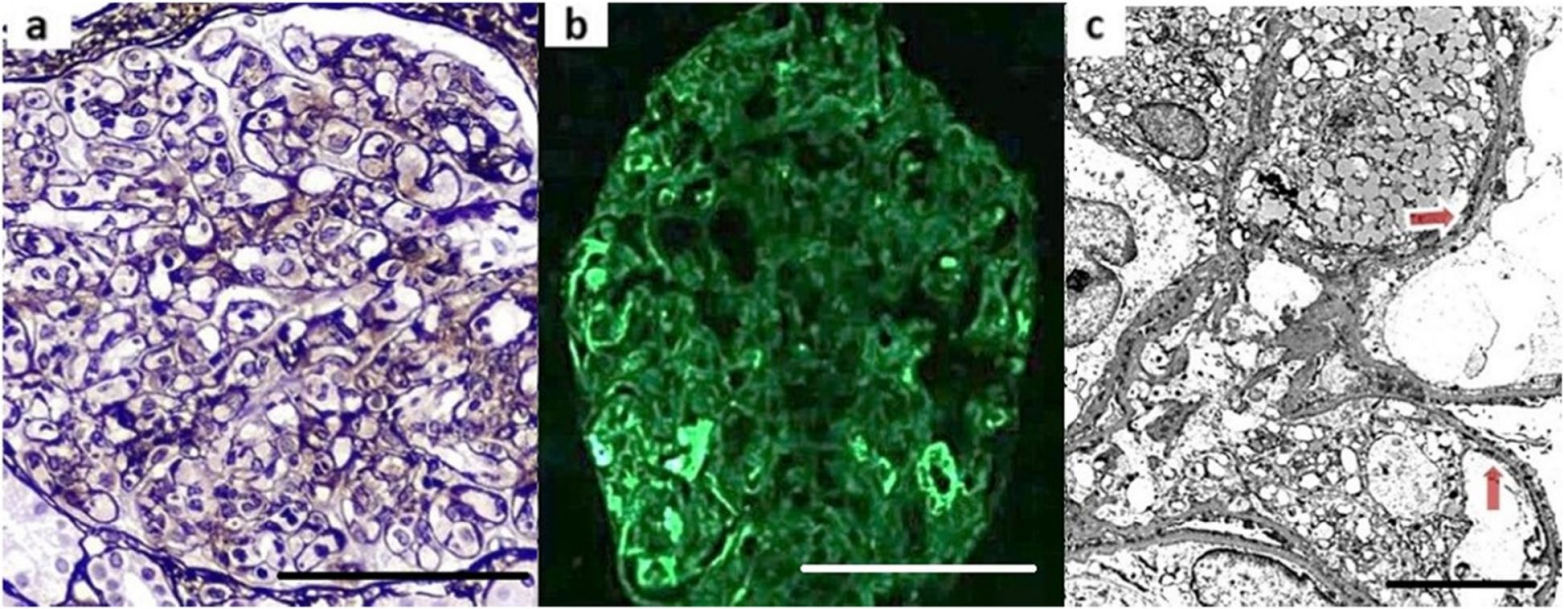
(First Kidney Biopsy). **a** Diffuse proliferative glomerulonephritis with endocapillary hypercellularity (Jones X 240). Bar, 100 μm. **b** IgM immunostain shows segmental capillary walls and mesangial positivity (IgM X240). Bar, 100 μm. 1c: EM showing intracapillary macrophages and few subendothelial deposits (arrows). (Original magnification x2000). Bar, 2.0 μm

A kidney biopsy was done at that time. It contained 22 glomeruli (3 of them globally sclerosed) which showed segmental duplication of capillary walls and endocapillary hypercellularity comprising mainly of mononuclear cells with fewer polymorphonuclear leukocytes (Fig. 1a). A single subcortical scar and few small cortical scars were seen populated by a sparse mononuclear cell infiltrate that was composed of CD3, CD20 and CD68 positive cells; CD5 and BCL2 were negative. Mild arteriosclerosis was seen. Immunofluorescence microscopy showed granular capillary wall positivity for C3 (1+), IgM (1+), kappa (2+) & lambda (1+). Mesangial C3 (1+) & IgG (traces) as seen. (Fig. 1b). Electron microscopy revealed podocytes with vacuolated cytoplasm and 90% effacement of their foot processes. There were segmental small electron dense deposits and interposition of lipid-laden mesangial cells in the subendothelial space. Few small electron densities were seen in an expanded mesangial matrix. Most capillary lumina were filled with macrophages & swollen endothelial with occasional polymorphonuclear leukocytes. Tubuloreticular inclusions were not detected (Fig. 1c).

She was started on Angiotensin Converting Enzyme (ACE) inhibitors and showed an improvement in proteinuria from 4.5 to 2.1 grams/24 hours, while her serum Albumin was 32 g/L (RR 38-50g/L). Her serum Creatinine was 80 μmol/L (RR of 49-90) and estimated glomerular filtration rate was 55 ml/min/1.73m^2^. The patient’s parameters remained stable on regular follow up. She was kept under observation for her indolent, stage 3 NMZL.

At this stage the clinical working diagnosis was NS with diffuse proliferative glomerulonephritis associated with scleroderma that was responding to conservative measures.

The patient presented 19 months later with 2 weeks history of progressive shortness of breath, dry cough, orthopnoea, paroxysmal nocturnal dyspnoea, uncontrolled HTN and worsening lower limb oedema with proteinuria of 4.7 g/24 hours and an increase in serum Creatinine levels from a baseline of 84 to 165 μmol/L.

A pleural tap yielded an exudate with reactive T-cell lymphocytosis and no malignant cells on flow cytometry. A pleural biopsy was unremarkable.

Urine Analysis revealed microscopic haematuria. Complements 3 value was 0.85 g/dL (RR 0.9-2.07 g/dL) and complement 4 value was 0.06 g/dL (RR 0.17-0.56 g/dL). Hepatitis B and C, cryoglobulins, Antineutrophil Cytoplasmic Antibodies (ANCA) and Antiphospholipase A2 Receptor (PLA2R) antibodies were all negative. ANA was repeated at the time of second biopsy and was again positive. On the other hand, Anti-DsDNA was done at the time of the first and second biopsy and were both negative.

A serum protein electrophoresis was reported as ‘a monoclonal gammopathy with an excess of free kappa light chains cannot be excluded’ (kappa/lambda ratio= 1.78; RR =0.26-1.65) with a simultaneous increase in free lambda light chain caused possibly by increased synthesis by an autoimmune process or due to renal damage (free kappa light chains= 62.7 mg/L; free lambda light chains= 35.2 mg/L).

**Fig. 2:**
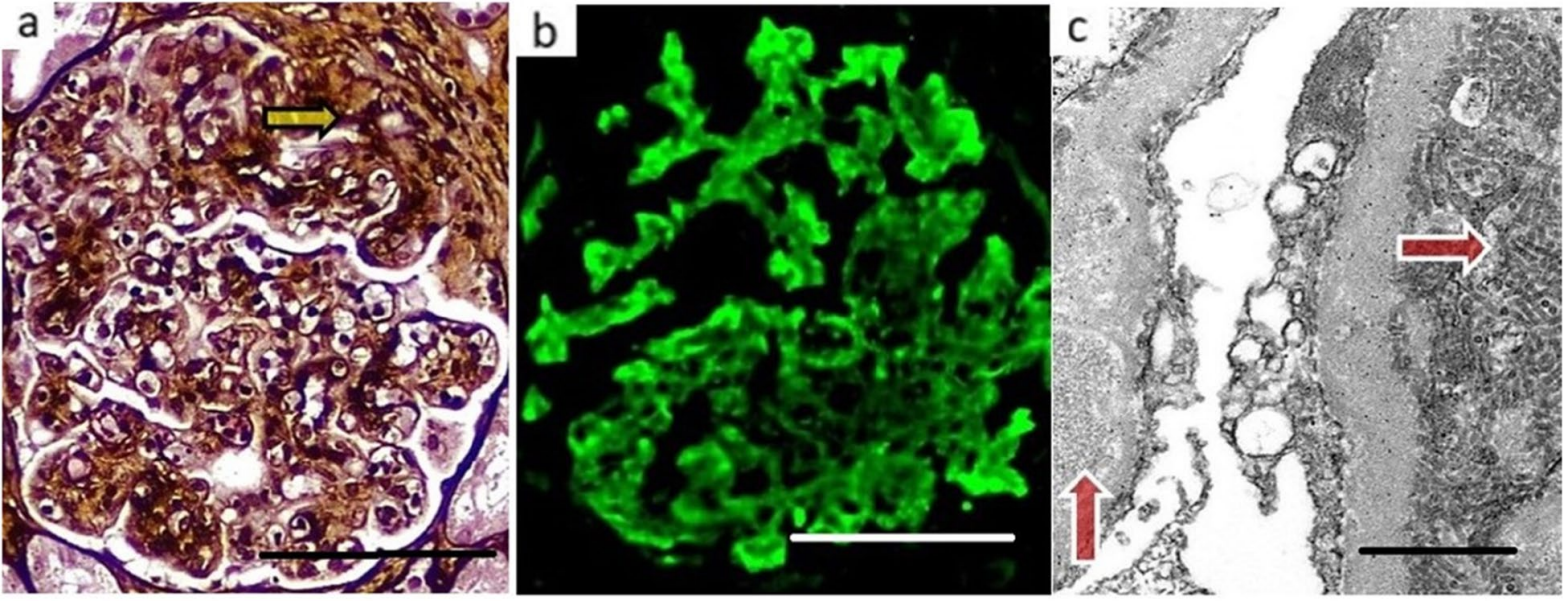
(Second Kidney Biopsy). **a** Membranoproliferative glomerulonephritis pattern with a cellular crescent (arrow). (Jones X240). Bar, 100 μm. **b** Kappa light chain stain decorates capillary walls & mesangium. (Kappa X240). Bar, 100 μm. **c** EM showing subendothelial deposit of microtubules (right arrow) and intramembranous deposit of thinner fibrils in an adjacent capillary loop (left arrow). (Original magnification x6000). Bar, 0.5 μm

Second kidney biopsy performed at this stage included 9 glomeruli, all perfused showing lobulated profiles with segmental duplication of glomerular basement membrane and endocapillary hypercellularity (an membranoproliferative glomerulonephritis pattern) and one segmental cellular crescent (Fig. 2a). Five small arteries showed severe fibrointimal thickening. Congo Red & Thioflavin T stains for amyloid were negative. Immunohistochemistry for lymphoid markers (CD3, CD5, CD20, CD43, CD68 and BCL2) showed no evidence of lymphoma. Immunofluorescence microscopy was positive for IgG (1+), IgM (2+) and C3 (2+) along capillary walls and mesangium. A positive staining for kappa light chain in a similar distribution, with a negative one for lambda, was consistent with kappa light chain restriction (Fig. 2b).

Electron microscopy revealed podocytes with marked cytoplasmic vacuolation and 90% effacement of their foot processes. There were groups of microtubules with hollow centres of a mean diameter of 39 nanometres mostly in a parallel arrangement in subendothelial spaces, while adjacent capillary loops displayed thinner, solid microfibrils with a mean diameter of 9 nm #in a mixed random and parallel arrangement in an intramembranous location (Fig. 2c). Mesangial areas showed an increase in mesangial matrix. Most capillary lumina were filled with macrophages & swollen endothelium with occasional polymorphonuclear leukocytes.

The kidney function continued to deteriorate with serum creatinine reaching a maximum of 315 μmol/L. with reduced urine output. The patient developed pulmonary oedema with significant volume overload, requiring intensive care unit admission with intubation and received few sessions of renal replacement therapy.

**Fig. 3:**
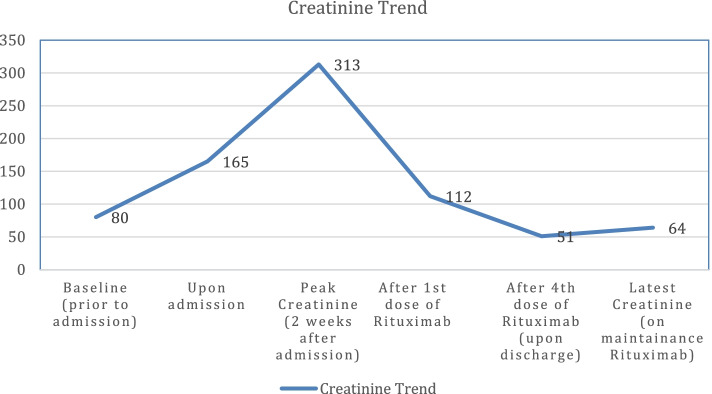
Graph showing the rise in creatinine from baseline to its peak level two weeks after admission and the dramatic improvement after initiating Rituximab

**Fig. 4:**
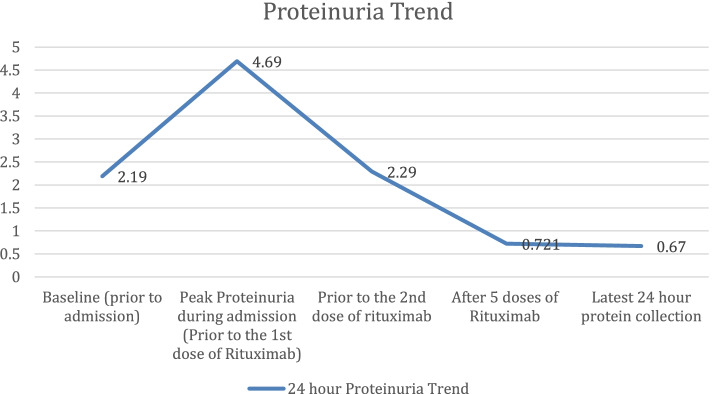
Graph showing the change in the 24 hour proteinuria from baseline and the improvement after initiating Rituximab

Decision was made to start the patient on rituximab at a dose of 375 mg/m^2^ weekly for four doses then to continue every 2 months. She was also given methylprednisolone 40 mg once daily intravenously for 1 week which was then tapered over 1 week. The kidney function gradually improved, leading to cessation of renal replacement therapy. She received a total of 4 doses of rituximab over a span of 6 weeks prior to discharge. Her ACE inhibitors were resumed. On discharge, her creatinine was back to normal (51 μmol/L) with a creatinine clearance of 87 ml/min/1.73 m^2^, serum albumin improved to 34 g/L with no evidence of volume overload and an improved urinary output with final discontinuation of diuretics (Figs 3 and 4).

After discharge, the patient continued the rituximab regimen every 2 months and had completed 1 year on maintenance therapy (a total of 6 maintenance doses to date). She will continue rituximab every 3 months for another year. The 24-hour urinary protein on follow up a year later was 0.67 g/24h with normal kidney function. No side effects were experienced from Rituximab.

## Discussion

ITG is a rare glomerulopathy encountered in 0.06-0.1% of native kidney biopsies [2, 3]. It has been recognized more frequently during the past 30 years and is characterised by glomerular deposits of microtubules with external diameters of 10-50 nm that appear to be composed of immunoglobulin and complement and are negative for amyloid by Congo Red stain [1]. Monoclonal immunoglobulins are detected by immunofluorescence microscopy in about 2/3^rd^ of the cases, usually with an underlying low grade lymphoid neoplasm of B-cell type (chronic lymphocytic lymphoma/leukaemia or multiple myeloma) [2, 3]. Rest of the cases are polyclonal often with autoimmune disorders [2].

NS is one of the common presentations of ITG [2, 3, 9]. Conversely, a retrospective study including cases from 1975 to 1994 assessing patients with NS showed ITG as the primary glomerular disease in 1% in black adults and 6% in white adults [10].

Patients with ITG can also present with haematuria, renal insufficiency and HTN and may proceed to ESRD [2, 3]. Our patient’s profile matched this with a decline in her renal function, HTN, oedema and worsening of proteinuria with hypoalbuminemia. Despite a previous history of NS, her proteinuria was always in the subnephrotic range (2.1 g/24 hours) prior to admission. On admission, her proteinuria worsened with significant oedema and hypoalbuminemia meeting the criteria of NS. At some point the patient became oliguric requiring renal replacement therapy temporarily.

An observational study in 1985 on 11 patients diagnosed with ITG showed progressive worsening of renal function in 6 patients, 5 of them requiring dialysis over 2 years’ of follow up, whereas 5 patients had stable renal function. It was noted patients with male gender, proteinuria of more than 3g/24 hours or severe HTN had a worse prognosis [11].

An association between monoclonal gammopathy or lymphoproliferative disorders and organized tubular deposits in the glomeruli as seen in ITG is recorded in literature; however, a relationship of either with NMZL has not been reported before [2, 3]. Nasr et al in their study of 73 cases of ITG published in 2020 found an underlying B-cell derived neoplasm as the commonest cause; furthermore, of the ten patients (14%) who had an underlying autoimmune disorder, none had scleroderma which was the co-morbidity in our case [2]. However, one online educational, non-peer reviewed case report has described scleroderma and ITG with a lambda light chain restriction [12]. For prognostic purposes it is important to differentiate between monoclonal and polyclonal types of ITG since the latter are more likely to proceed to ESRD [2, 3].

MZL are mainly seen in the elderly: they comprise 26% of non-Hodgkin’s lymphomas in over 80s and NMZL represent 10% of MZL [6]. A monoclonal gammopathy exists in 10% of NMZL cases [13].

At this stage it is pertinent to note here that an association between a variety of autoimmune diseases and MZL is also well-recognised [7]. Thus, the rare occurrence of MZL in cases of systemic sclerosis was reported in a review on this subject [8].

It is also relevant that, while MZL have been reported in systemic sclerosis, ours is the first case where a possible association between SSSS and NMZL is recorded and evolution of ITG in this setting is documented.

The adoption of kappa/lambda ratio as a marker of monoclonal gammopathy rather than an absolute excess of serum levels of free kappa or lambda chains reflects the fact that raised levels of individual free light chains maybe a feature of polyclonal hypergammaglobulinaemias as in autoimmune disorders or impaired kidney functions [14]. In our case, the demonstration of kappa light chain restriction by immunofluorescence microscopy is consistent with a diagnosis of monoclonal gammopathy, although free light chains of both monoclonal and polyclonal origins were also possibly present.

The approximate duration from the diagnosis of NMZL to the diagnosis of ITG in our patient was two years. During this time, the patient was kept on regular follow up with haematology department, and there were no clear indications to start treatment as our patient was asymptomatic and there was no progression of her lymphoma. Therefore, she was kept on close observation. Perhaps, if the lymphoma was treated at the time of the diagnosis, the development of ITG could have been prevented. In the study published by Nasr SH et al, the underlying hematologic conditions were discovered 14-168 months before ITG in half of patients with available data, and concomitantly in the remaining patients [2]. Our dilemma was to decide if the ITG was related to a NMZL or SSSS. In this regard it is worth noting that in our patient the discovery of ITG in the second biopsy was concomitant with detection of excess free kappa light chains in serum protein electrophoresis/immunofixation that suggests emergence of a clone within the NMZL. We speculate that the NMZL evolved in a setting of SSSS and subsequent emergence of a free kappa light chain secreting clone in the interval between the 2 biopsies led to the glomerular deposition of microtubular structures typical of ITG. One important weakness of this scenario is that Electron Microscopy of the first biopsy may have missed early microtubular deposits since the samples for that study are usually minute. Another possibility is that NMZL was solely responsible for ITG with SSSS being a mere co-morbidity.

The mechanism underlying the genesis of distinctive microtubules is not fully understood, however the demonstration of these structures within the cytoplasm of malignant lymphoid cells and the recurrence of these deposits in the allografts suggest that these are deposited in the glomeruli rather than produced in situ [3].

Rituximab is the one of contemporary treatments used in ITG. A recent cohort study assessed the effect of rituximab or bortezomib-based chemotherapy was studied on 21 patients. It was noted that 16 out of 21 sustained a renal response after 40-month follow up [3]. This corroborates with our experience with rituximab therapy that led to a sustained remission to date.

Two large studies on ITG have shown that in patients with underlying haematological malignancies, the prognosis is highly dependent on successful management of the malignant B-cell clone that we have achieved with rituximab therapy [2, 3].

## Conclusion

In conclusion, ITG is one of the rare glomerulopathies characterised by glomerular deposition of microtubules composed of immunoglobulin and complement. One of the prevailing treatments used in the management of ITG with lymphoid neoplasia is rituximab, which shows a promising effect in reversing the adverse events of the disease as it targets the underlying neoplastic B-cell proliferation. In this case study we have documented the evolution of ITG in the setting of NMZL and SSSS in a 79-year-old female patient over a 19 months period. She was treated with rituximab with remarkable and sustained improvement of her kidney function and proteinuria.

## Data Availability

Raw data supporting the findings of this case report as well as all datasets generated and analyzed during the current study are available from the corresponding author on reasonable request.

## Abbreviations

ANCA: Antineutrophil Cytoplasmic Antibodies
ESRD: End Stage Renal Disease
HTN: Hypertension
Ig: Immunoglobulin
ITG: Immunotactoid Glomerulopathy
MZL: Marginal Zone Lymphoma
NMZL: Nodal Marginal Zone Lymphoma
NS: Nephrotic Syndrome
RR: Reference Range
SSSS: Systemic Sclerosis Sine Scleroderma
